# Phylogenomic reclassification of the world’s most venomous spiders (Mygalomorphae, Atracinae), with implications for venom evolution

**DOI:** 10.1038/s41598-018-19946-2

**Published:** 2018-01-26

**Authors:** Marshal Hedin, Shahan Derkarabetian, Martín J. Ramírez, Cor Vink, Jason E. Bond

**Affiliations:** 1 Department of Biology, San Diego State University, San Diego, CA 92182–4614 USA; 2 Department of Biology, University of California Riverside, Riverside, California 92521 USA; 3 Division of Arachnology Museo Argentino de Ciencias Naturales “Bernardino Rivadavia” Consejo Nacional de Investigaciones Científicas y Técnicas (CONICET) Ciudad Autónoma de Buenos Aires, Buenos Aires, Argentina; 4 Canterbury Museum Christchurch, Christchurch, 8013 New Zealand; 5 Department of Biological Sciences, Auburn University, Auburn, AL 36849 USA

## Abstract

Here we show that the most venomous spiders in the world are phylogenetically misplaced. Australian atracine spiders (family Hexathelidae), including the notorious Sydney funnel-web spider *Atrax robustus*, produce venom peptides that can kill people. Intriguingly, eastern Australian mouse spiders (family Actinopodidae) are also medically dangerous, possessing venom peptides strikingly similar to *Atrax* hexatoxins. Based on the standing morphology-based classification, mouse spiders are hypothesized distant relatives of atracines, having diverged over 200 million years ago. Using sequence-capture phylogenomics, we instead show convincingly that hexathelids are non-monophyletic, and that atracines are sister to actinopodids. Three new mygalomorph lineages are elevated to the family level, and a revised circumscription of Hexathelidae is presented. Re-writing this phylogenetic story has major implications for how we study venom evolution in these spiders, and potentially genuine consequences for antivenom development and bite treatment research. More generally, our research provides a textbook example of the applied importance of modern phylogenomic research.

## Introduction

*Atrax robustus*, the Sydney funnel-web spider, is often considered the world’s most venomous spider species^1^. The neurotoxic bite of a male *A*. *robustus* causes a life-threatening envenomation syndrome in humans. Although antivenoms have now largely mitigated human deaths, bites remain potentially life-threatening^2^. *Atrax* is a member of a larger clade of 34 described species, the mygalomorph subfamily Atracinae, at least six of which (*A*. *robustus* and five *Hadronyche* species) cause severe envenomation in humans^3^. The venoms of a handful of assayed atracines include a δ-hexatoxin that induces delayed inactivation of voltage-gated sodium channels in primates^4,5^. Atracine venoms also include insect-specific inhibitor cystine knot (ICK) neurotoxins^6^ that have been proposed as natural bioinsecticides^5,7,8^. Chassagnon *et al*.^9^ recently showed that a unique *Hadronyche* double-knot venom peptide shows therapeutic potential in protecting the human brain from damage after stroke events.

**Figure 1:**
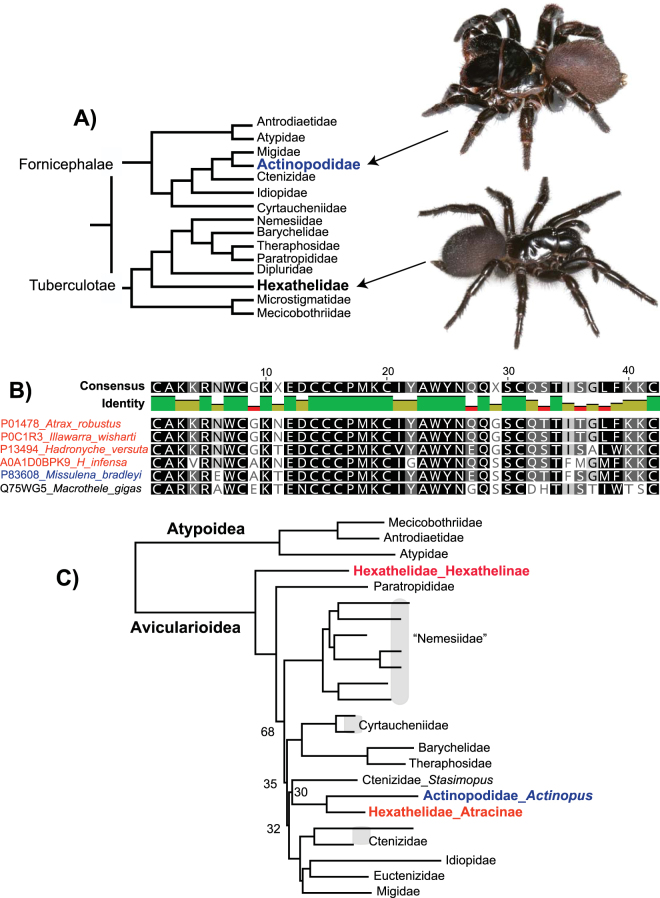
(**A**) Summary of Raven^12^ phylogeny and currently accepted family-level classification of mygalomorph spiders (except for new family Euctenizidae^13^), with distant placement of hexathelids and actinopodids highlighted. Taxonomic names follow Raven^12^. Images of live female *Missulena sp*. and *Atrax robustus*. (**B**) *Missulena* and *Atrax* δ-hexatoxin homology. Results based on UniProt BLASTP search of mature δ-hexatoxin-Ar1a. (**C**) Summary of Hamilton *et al*.^19^ phylogeny, based on concatenated RAxML analysis of 327 anchored hybrid enrichment loci. Bootstrap = 100 if not shown.

Atracinae includes three genera (*Atrax*, *Illawarra*, and *Hadronyche*^10,11^), found in eastern and southern Australia, currently placed in the family Hexathelidae. Based on the currently accepted classification [following refs^12,13^], hexathelids are distantly related to actinopodid mygalomorphs (Fig. 1A), an austral family that includes Australian mouse spiders (*Missulena*). Significantly, *Missulena* venom has a mode of action similar to that of *Atrax*, includes peptides clearly homologous to δ-hexatoxins (Fig. 1B), and *Missulena* bites are treated effectively using *Atrax* antivenoms^14–16^. Gunning *et al*.^15^ proposed that the similarities observed between *Atrax* and *Missulena* venoms “*provides evidence of a highly conserved spider N-toxin from a phylogenetically distinct spider family that has not undergone significant modification*”. This “ancient conservation” hypothesis implies a broad phylogenetic distribution of potentially dangerous venom proteins in mygalomorph spiders (Fig. 1A), although an alternative is convergent evolution at the protein level in distant relatives.

We instead hypothesize that *Atrax* and *Missulena* venom similarities reflect homology from more recent shared ancestry (“recent homology” hypothesis). An atracine plus actinopodid relationship has been suggested in multiple molecular phylogenetic studies [refs^13,17–20^; Fig. 1C], all of which were hindered by a small and incomplete sample of hexathelids and actinopodids. Here we test the recent homology hypothesis using phylogenomic analyses of ultraconserved element (UCE) sequences for a taxon sample that includes all described hexathelid and actinopodid genera, and a relevant sample of other mygalomorph genera. We show convincingly that hexathelids are not monophyletic, and that atracines are sister to austral actinopodids. This result has significant implications for mygalomorph family-level classification, and for the study of venom evolution in these medically and economically important spiders.

## Results and Discussion

We sampled all described hexathelid and actinopodid genera^21^. Many of these genera are geographically restricted and rare (e.g., *Plesiothele* from isolated highlands in Tasmania, *Plesiolena* from a handful of specimens from remote Chile), thus requiring the use of standard museum specimens for DNA extraction from some taxa (see Methods). In addition, we sampled atypoids as outgroups, and multiple diplurid genera, following hypothesized affinities of hexathelids with diplurids^12,13,18,22,23^. Although we did not generate UCE data for representatives of all mygalomorph families, there are no genera missing from our sample that are clear close atracine or actinopodid relatives, as suggested by recent molecular phylogenetic studies^13,19,20^.

**Figure 2:**
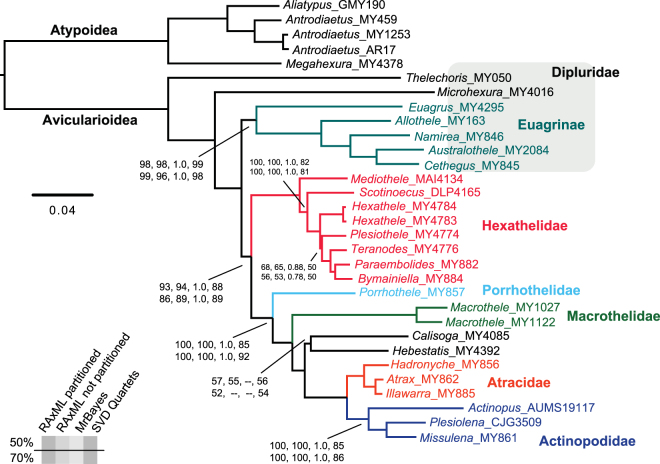
Partitioned RAxML concatenated phylogeny, based on 70% occupancy matrix. Support values from other analyses shown. If support values not shown, support = 100 or 1.0. *Calisoga* plus *Hebestatis* sister relationship is poorly supported, in some phylogenies recovered as (*Hebestatis*, (*Calisoga*, (atracids  + actinopodids))).

We analyzed both 50% (514 loci, 101652 basepairs) and 70% occupancy (381 loci, 78103 basepairs) UCE matrices (Supplemental Table 1). The following pertinent clades were recovered with full support (bootstrap = 100, posterior probability = 1.0) in all phylogenomic analyses, regardless of method or model used: Avicularioidea (non-atypoids with male bulb sclerites fused, lacking abdominal sclerites, etc.), Hexathelinae (including *Plesiothele*), Atracinae, and an atracine plus actinopodid clade (Fig. 2, Supplemental Fig. 1). Hexathelids are always fragmented into four distinct lineages, below reclassified as four separate families. As such, austral biogeographic patterns (southern South America + Australia/New Zealand) are independently replicated in the hexatheline and actinopodid lineages. Concatenated and coalescent methods are consistent in the recovery of major clades. One notable difference is the ASTRAL placement of the *Porrhothele* plus relatives clade, but this placement is weakly supported in ASTRAL analyses (Fig. 2, Supplemental Fig. 1).

Many mygalomorph species are short-range endemics, known from few locations, often from very few (<10) specimens. In addition, these spiders often live notoriously cryptic lifestyles, residing in difficult-to-find subterranean burrows, concealed by hidden trapdoors or other entrance constructs. Basically all previous molecular phylogenetic studies have been somewhat hindered by this rareness and/or cryptic biology, with taxa excluded because “DNA-preserved” samples were unavailable. Here we were able to generate 100 s of UCE loci from specimens collected over 30 years ago, and subsequently preserved in low percentage alcohol at room temperatures (i.e., “standard” museum specimens). It is important to note that only museum specimens extracted using phenol/chloroform resulted in useable data; four older specimens extracted using Qiagen all failed (Supplemental Table 1). UCE-based phylogenomics from museum specimens has been demonstrated for other animal taxa [e.g., bees^24^, birds^25^, snakes^26^,]. Our study extends this utility to arachnids, and demonstrates the potential effectiveness of the UCE method for thousands of rare taxa currently residing in museums worldwide.

Detailed comparisons to earlier studies of mygalomorph phylogeny are provided in the Supplemental Text. Here we make three general claims that are supported by this and prior studies. First, hexathelids, defined by a single morphological synapomorphy (possession of numerous labial cuspules^12,27^), are not monophyletic and require re-classification. Second, hexathelines (with numerous labial cuspules and six spinnerets) are relatively early-diverging avicularioids, along with multiple non-diplurine diplurid lineages (e.g., Ischnothelinae, Euagrinae, etc.). Third, atracine hexathelids are monophyletic, and sister to a monophyletic Actinopodidae. This combined lineage occupies a relatively derived position in mygalomorph phylogeny [see also ref.^19^]. A much larger phylogenomic sample including multiple representatives of all mygalomorph families will be required to solidify this placement.

Like other spiders, atracine venoms are complex chemical cocktails, including a very large number of peptides and other molecules^7^. For example, Palagi *et al*.^28^ used modern mass spectrometry methods to survey venoms of multiple atracine taxa and found a large number of peptides (800 peptides in female venoms, ~400 in male venoms), marked sexual differences, and clear species-level differences. Despite this peptide diversity, primate-targeting δ-hexatoxins are a primary component of the atracine venom peptidome^4^, with some species possessing multiple δ-hexatoxin in-paralogs^28^. Even with minor differences at the protein level (Fig. 1B), bites of all atracines with these δ-hexatoxins cause a superficially similar envenomation syndrome in humans^4,29^. Among known spider venom peptides, the δ-actinopoditoxin of male *Missulena bradleyi* is most similar to atracine δ-hexatoxins (Fig. 1B), and *M*. *bradleyi* venoms have a similarly selective mode of action on vertebrate sodium channels^14,15^. Furthermore, *Missulena* bites are sometimes of medical concern^16,30^, and such bites are effectively treated using antivenoms developed for atracines^14^. Our phylogenomic results (Fig. 2) indicate that all of these biological similarities reflect recent shared common ancestry of these spider lineages, rather than the alternatives of convergence or ancient phylogenetic conservation of venom composition.

Our phylogenomic hypothesis (Fig. 2) provides a robust comparative framework for addressing the evolutionary assembly of venoms, including the medically important δ-hexatoxins, in the atracine plus actinopodids clade. We make the following general predictions. First, we hypothesize that both *Actinopus* and *Plesiolena* possess homologs of δ-hexatoxins. Characterization of *Actinopus* venom peptides in particular would allow reconstruction of ancestral proteins for the entire clade. Our prediction also implies that both *Actinopus* and *Plesiolena*, like *Missulena*, have the potential to cause dangerous bites. We note however that Mullen and Vetter^31^ state that *Actinopus* bites in southern South America “produce only local pain and transient muscle contractions”. Our phylogenomic hypothesis indicates that the most relevant taxon for understanding *Atrax* venom evolution is the little-studied sister genus *Illawarra*, as also reflected by very high δ-hexatoxin similarity (Fig. 1B). Finally, we note that the species tree framework specified here provides a basis for the study of all venom molecules in these spiders, such as the insect-specific ICK neurotoxins in the Shiva superfamily^6^. A comprehensive study of the venom peptidome in all atracine plus actinopodid genera would provide considerable insight into molecular evolution in these important spiders.

### Taxonomy

Here we summarize the revised taxonomy of the Hexathelidae and related new familial rank taxa; all nomenclatural changes proposed are to be attributed to Hedin and Bond. The subfamily Atracinae is removed from Hexathelidae and elevated to the rank of family (NEW RANK); it includes the genera listed below. The subfamily Macrothelinae (Simon, 1892) is removed from Hexathelidae and elevated to the rank of family (NEW RANK). The genus *Porrhothele* is removed from Hexathelidae (subfamily Macrothelinae) and designated as a family (NEW FAMILY). The revised circumscription of the family Hexathelidae is documented below.

### Family Atracidae Hogg, 1901 (NEW RANK)

#### Type genus

*Atrax* O. Pickard-Cambridge, 1877 (type species *Atrax robustus* O. Pickard-Cambridge, 1877).

#### Remarks

Atraceae, originally described by Hogg^32^, comprised the two genera *Atrax* and *Hadronyche*. The group was subsequently formally designated as a subfamily by Gray^10^ and diagnosed on the basis of taxa having “a broad embolic shaft” (males) and having two rows of large cheliceral teeth along with distinctive leg spination (spines on the tarsi), numerous labial cuspules, and a “coniform” anterior endite lobe. Gray^11^ provided a more thorough diagnosis and description of the subfamily along with a new circumscription to include the genus *Illawarra*. Atracidae are found in Australia (Queensland, New South Wales, Australian Capital Territory, Victoria, South Australia and Tasmania).

### List of included genera

*Atrax* O. Pickard-Cambridge, 1877 [urn:lsid:nmbe.ch:spidergen:00013]

*Hadronyche* L. Koch, 1873 [urn:lsid:nmbe.ch:spidergen:00015]

*Illawarra* Gray, 2010 [urn:lsid:nmbe.ch:spidergen:03995].

### Family Macrothelidae (Simon, 1892) (NEW RANK)

#### Type genus

*Macrothele* Ausserer, 1871 [urn:lsid:nmbe.ch:spidergen:00017] (type species *Macrothele calpeiana* Walkenaer, 1805).

#### Remarks

As a consequence of its monotypy, characters used to diagnose the family Macrothelidae are those characters attributed to the type genus. Per Raven^27^ macrothelids can be diagnosed from other mygalomorph taxa by having much larger posterior sternal sigilla and a single row of larger teeth on the cheliceral promargin with smaller teeth on the retromargin. As with other taxa discussed in this study, a more careful examination of morphology is warranted in light of these changes. Although monogeneric families are not ideal, it should be noted that members of the genus *Macrothele* are consistently recovered as a distinct, relatively early-diverging avicularioid lineage (e.g., see^18^; Supplemental Text). This monotypic family is known from Africa, Asia and parts of Europe (Spain, Italy, Greece).

### Family Porrhothelidae (NEW FAMILY)

#### Type genus

*Porrhothele* Simon, 1892 [urn:lsid:nmbe.ch:spidergen:00021] (type species *Porrothele* *antipodiana* (Walkenaer, 1837).

#### Diagnosis

As a consequence of its monotypy, characters used to diagnose the family Porrhothelidae are those characters attributed to the type genus. *Porrhothele* was thoroughly diagnosed and described by Forster^33^ with additions by Raven^27^. Members of this family can be diagnosed on the basis of the following unique combination of characters: 1) small posterior sternal sigilla; 2) single row of promarginal cheliceral teeth; 3) male tibia swollen with dense pattern of strong promarginal spines (illustrated in Forster;^33^ e.g., p. 170, figs. 543–548). This monotypic family is found in New Zealand.

### Family Hexathelidae (Simon, 1892) (new circumscription)

#### Type genus

*Hexathele* Ausserer, 1871 [urn:lsid:nmbe.ch:spidergen:00016] (type species *Hexathele hochstetteri* Ausserer, 1871)

Subfamily Hexathelinae (Simon, 1892)

Plesiothelinae Raven 1980. Type genus *Plesiothele* Raven, 1978 (type species *Plesiothele fentoni* (Hickman, 1936)). New synonymy.

#### Remarks

Hexathelidae is found in Australia (Queensland, New South Wales, Australian Capital Territory, Victoria, Tasmania), New Zealand, Chile and Argentina.

### List of included genera

*Bymainiella* Raven, 1978 [urn:lsid:nmbe.ch:spidergen:00014]

*Hexathele* Ausserer, 1871 [urn:lsid:nmbe.ch:spidergen:00016]

*Mediothele* Raven & Platnick, 1978 [urn:lsid:nmbe.ch:spidergen:00018]

*Paraembolides* Raven, 1980 [urn:lsid:nmbe.ch:spidergen:00019]

*Plesiothele* Raven, 1978 [urn:lsid:nmbe.ch:spidergen:00020]

*Scotinoecus* Simon, 1892 [urn:lsid:nmbe.ch:spidergen:00022]

*Teranodes* Raven, 1985 [urn:lsid:nmbe.ch:spidergen:00023]

## Methods

### Specimen sampling and DNA extraction

A majority of specimens were personally identified by the authors, and are vouchered at SDSU, Auburn, and Museo Argentino de Ciencias Naturales **(**Supplemental Table 1). Most specimens were preserved for DNA studies (preserved in high percentage ethyl alcohol at −80 °C), and genomic DNA was extracted from leg tissue using the Qiagen DNeasy Blood and Tissue Kit (Qiagen, Valencia, CA). Standard phenol/chloroform extractions with 24-hour incubation for lysis were used for three samples (*Mediothele nahuelbuta* MAI4134, *Plesiolena bonnetti* CJG3509, *Scotinoecus* sp. DLP4165; Supplemental Table 1), as tissues were preserved in 70–80% EtOH and initial Qiagen extractions produced no quantifiable DNA. All extractions were quantified using a Qubit Fluorometer (Life Technologies, Inc.) and quality was assessed via gel electrophoresis on a 1% agarose gel. Qiagen extractions resulted in >500 ng for library preparation, while phenol/chloroform extractions of older material resulted in 26–226 ng total (Supplemental Table 1). UCE data for seven taxa were generated by Starrett *et al*.^34^.

### UCE data collection & matrix assembly

Sequence capture data were collected in multiple library preparation and sequencing experiments that differed mainly in sequencing platform. Up to 500 ng of genomic DNA was used in sonication, using either a Bioruptor for 7 cycles at 30 s on and 90 s off, or using a Covaris M220 Focused-ultrasonicator with treatment time of 65 s, Peak Incident Power of 50, 10% Duty Factor, and 200 cycles per burst. Samples were run out on agarose gels to verify sonication success.

Library preparation followed Starrett *et al*.^34^, with some modifications. Briefly, libraries were prepared using the KAPA Hyper Prep Kit (Kapa Biosystems), using up to 250 ng DNA (i.e., half reaction of manufacturer’s protocol) as starting material. Ampure XP beads (Beckman Coulter) were used for all cleanup steps. For samples containing <250 ng total, all DNA was used in library preparation. After end-repair and A-tailing, universal adapters were ligated onto libraries. Libraries were then amplified in a 25 μl reaction, with 15 μl adapter-ligated DNA, 25 μl 1 × HiFi HotStart ReadyMix, and 2.5 μl of each Illumina TruSeq dual-indexed primer (i5 and i7) with modified 8-bp indexes^35^. Amplification conditions were 98 °C for 45 s, then 16 or 18 cycles of 98 °C for 15 s, 60 °C for 30 s, and 72 °C for 60 s, followed by a final extension of 72 °C for 60 s. Samples were quantified again to ensure amplification success. Equimolar amounts of libraries were combined into 1000 ng total pools consisting of eight samples each (125 ng per sample).

Target enrichment was performed on pooled libraries using the MYbaits Arachnida 1.1 K version 1 kit (Arbor Biosciences^36^;) following the Target Enrichment of Illumina Libraries v. 1.5 protocol (http://ultraconserved.org/#protocols). Hybridization was conducted at 60 or 65 °C for 24 hours. Following hybridization, pools were amplified in a 50 μl reaction consisting of 15 μl of hybridized pools, 25 μl Kapa HiFi HotStart ReadyMix, 5 μl dH20, and 5 μM of each of TruSeq forward and reverse primers. Amplification conditions consisted of 98 °C for 45 s, then 16 or 18 cycles of 98 °C for 15 s, 60 °C for 30 s, and 72 °C for 60 s, followed by a final extension of 72 °C for 5 minutes. Following an additional cleanup, libraries were quantified using a Qubit fluorometer and equimolar mixes were prepared for sequencing either with an Illumina NextSeq (University of California, Riverside Institute for Integrative Genome Biology) or an Illumina HiSeq. 2500 (Brigham Young University DNA Sequencing Center).

Raw demultiplexed reads were processed with the Phyluce pipeline^37^. Quality control and adapter removal were conducted with the Illumiprocessor wrapper^38^. Assemblies were created with Velvet^39^ and Trinity^40^, both at default settings. Contigs from both assemblies were combined for probe matching, retrieving assembly-specific UCEs and overall increasing the number of UCEs per sample relative to using only a single assembly method. Contigs were matched to probes using minimum coverage and minimum identity values of 65. UCE loci were aligned with MAFFT^41^ and trimmed with Gblocks^42,43^ implemented in the Phyluce pipeline.

Individual UCE loci were imported into Geneious 10.1 (Biomatters Ltd.) and manually inspected. In particular, alignments with low % identical sites (less than 40%) were flagged for inspection. If exclusion of a single divergent sequence increased this value to >60%, the locus was retained. Subsequently, all loci were inspected - individual sequences with large internal (in conserved UCE region) gaps were excluded, and obvious alignment errors were manually adjusted. Finally, individual RAxML^44^ gene trees were reconstructed, with those loci not recovering a monophyletic Atypoidea excluded (taken as evidence for paralogy).

### Phylogenomic analyses

Two datasets were assembled for phylogenomic analyses, differing in the minimum taxon coverage (50% and 70%) needed for a locus to be included in the final dataset. Concatenated and partitioned maximum likelihood and Bayesian analyses were run for each dataset. Maximum likelihood analyses were conducted using RAxML version 8.2^44^ with the GTRGAMMA model and 200 rapid bootstrap replicates. Bayesian analyses were conducted with MrBayes 3.2.6^45^ on the CIPRES portal^46^. Analyses were run for 10 million generations, logging every 1000 generations. For partitioned analyses, partitions and models were chosen using PartitionFinder 1.1.1^47^. Two coalescent analyses were also conducted for both datasets. First, ASTRAL-II^48,49^ was used with individual gene trees estimated in RAxML with 500 bootstrap replicates. We also used SVDquartets^50,51^ with n = 500 bootstraps, as implemented in PAUP* 4.0^52^.

### Data availability

The datasets generated and analyzed during the current study are available from the NCBI Short-Read Archive (raw sequence reads, BioProject PRJNA423032) and Dryad (aligned matrices and. tre files, doi:10.5061/dryad.8d638).

## Electronic supplementary material

Supplemental Information

Supplemental Table 1
