# Supplementary material for: Phylogenomic reclassification of the world’s most venomous spiders (Mygalomorphae, Atracinae), with implications for venom evolution
Source: Sci Rep. 2018 Jan 26;8:1636. doi: 10.1038/s41598-018-19946-2 (PMC5785998; doi:10.1038/s41598-018-19946-2)
Supplement: Supplementary file 1 — Supplemental Information [file 41598_2018_19946_MOESM1_ESM.pdf]

Phylogenomic reclassification of the world's most venomous spiders (Mygalomorphae, Atracinae), with implications for venom evolution

Marshal Hedin, Shahan Derkarabetian, Martín J. Ramírez, Cor Vink, Jason Bond

Supplemental Information

Supplemental Text. Summary of prior hexathelid phylogeny and classification studies.

Although our overall results contrast with earlier morphology-only studies of mygalomorph relationships, several points of agreement can be found with more recent molecular studies. Raven [12, 27] re-limited the family Hexathelidae, and identified one morphological synapomorphy (possession of numerous labial cuspules). Raven [12] discussed at length the uncomfortable placement of *Atrax* (then the only described atracine), including hypothesized morphological homoplasy with more derived mygalomorph taxa. Goloboff [22] conducted a formal cladistic analysis of morphology, and recovered a paraphyletic Hexathelidae as an early-diverging Avicularioidea lineage, but noted that near parsimonious trees included hexathelids as monophyletic. Using ribosomal DNA sequences, Hedin & Bond [23] found a paraphyletic Hexathelidae, with taxa intermixed with non-diplurine diplurids at the base of Avicularioidea. Sampled atracines were not recovered with the single sampled actinopodid. Using elongation factor-1 gamma sequences, Ayoub *et al.* [17] recovered a *Hadronyche* plus *Missulena* clade, separate from the hexatheline *Bymainiella*. Using multiple genes alone, or molecules plus morphology, Bond *et al.* [13] recovered *Atrax* + *Hadronyche* with *Missulena* + *Actinopus* (F Actinopodidae). Again, hexathelids were not monophyletic, with two hexathelines intermixed with early-diverging non-diplurine diplurids. Opatova and Arnedo [18] used the Bond *et al.* [13] multigenic matrixes, adding data for multiple *Macrothele* specimens. These authors found four separate hexathelid lineages (i.e., hexathelid non-monophyly), a monophyletic *Macrothele*, and an atracine plus actinopodid sister relationship. Using a 6-gene dataset, Wheeler *et al.* [20] recovered a core hexathelid lineage (including the important South American taxa *Mediothele* and *Scotinoecus* as sister taxa), separate from a well-supported atracine plus actinopodid clade. Finally, Hamilton *et al.* [19] used anchored hybrid enrichment (AHE) sequence capture data to show hexathelid non-monophyly, and an atracinae plus actinopodid relationship (see **FIG 1C**).

Supplemental Table 1. Excel file with voucher, identification and UCE information.

Supplemental Figure 1. ASTRAL phylogenies.

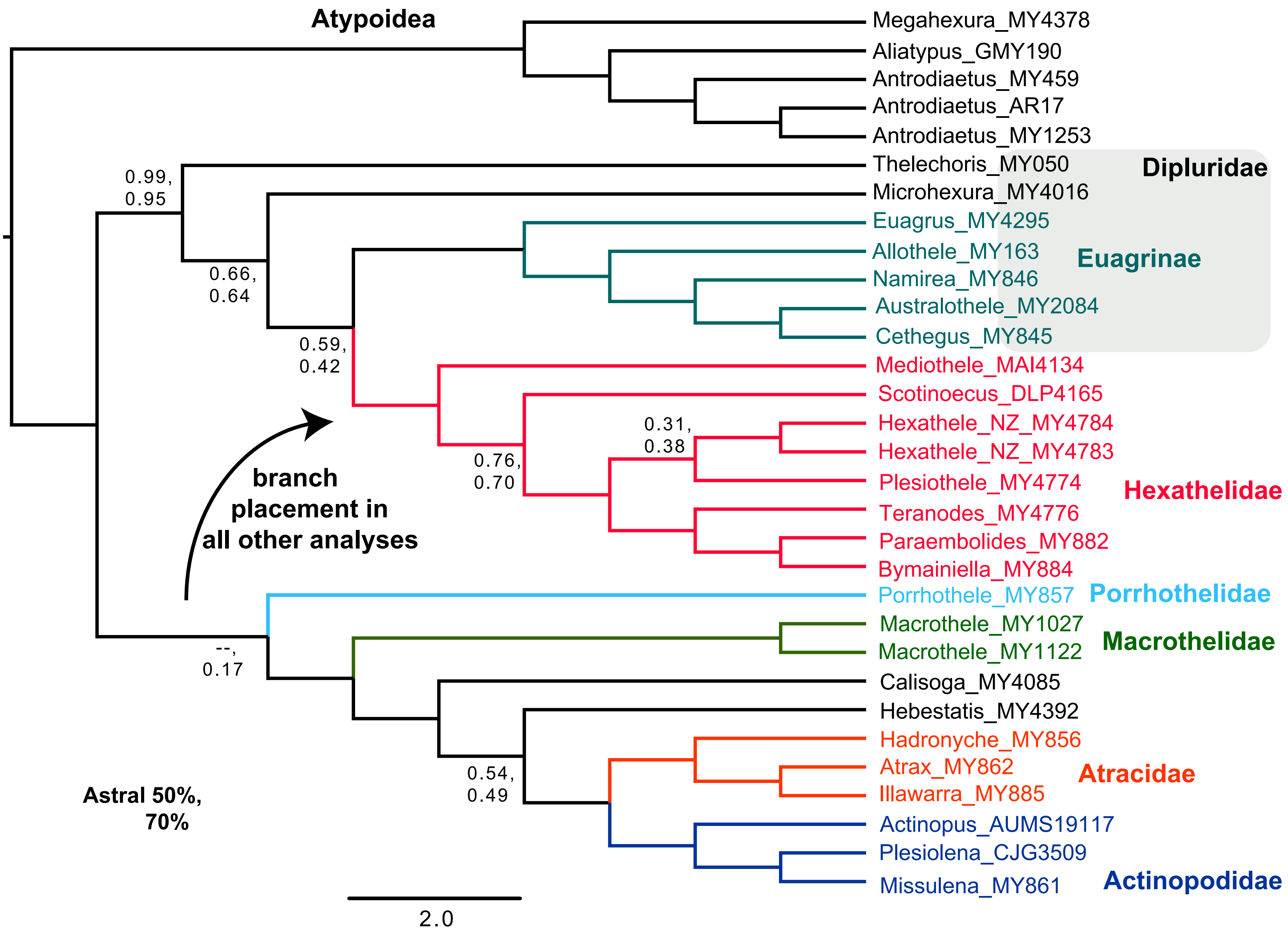
Atypoidea
Megahexura_MY4378
Aliatypus_GMY190
Antrodiaetus_MY459
Antrodiaetus_AR17
Antrodiaetus_MY1253
Thelechoris_MY050
Dipluridae
Microhexura_MY4016
Euagrus_MY4295
Allothele_MY163
Euagrinae
Namirea_MY846
Australothele_MY2084
Cethegus_MY845
Mediothele_MAI4134
Scotinoecus_DLP4165
Hexathele_NZ_MY4784
Hexathele_NZ_MY4783
Plesiothele_MY4774
Hexathelidae
Teranodes_MY4776
Paraembolides_MY882
Bymainiella_MY884
Porrhothele_MY857
Porrhothelidae
Macrothele_MY1027
Macrothele_MY1122
Macrothelidae
Calisoga_MY4085
Hebestatis_MY4392
Hadronyche_MY856
Atrax_MY862
Illawarra_MY885
Atracidae
Actinopus_AUMS19117
Plesiolena_CJG3509
Missulena_MY861
Actinopodidae
0.99,
0.95
0.66,
0.64
0.59,
0.42
0.76,
0.70
0.31,
0.38
--,
0.17
0.54,
0.49
branch
placement in
all other analyses
Astral 50%,
70%
2.0
